# Health Promoting Schools Provide Community-Based Learning Opportunities Conducive to Careers in Rural Practice

**DOI:** 10.1155/2011/892518

**Published:** 2011-04-07

**Authors:** Andrew Macnab, Arabat Kasangaki, Faith Gagnon

**Affiliations:** ^1^Department of Pediatrics, University of British Columbia, Vancouver, BC, Canada V6H 3V4; ^2^Wallenberg Research Centre, Stellenbosch Institute for Advance Study (STIAS), Stellenbosch University, Stellenbosch 7600, South Africa; ^3^College of Health Sciences, Makerere University, P.O. Box 7062, Kampala, Uganda; ^4^Gagnon Research Associates, Surrey, BC, Canada V4A 1T7

## Abstract

The World Health Organization conceived “health-promoting schools” as a means of providing the information and support systems necessary for the worldwide changes in behavior that are needed to improve health globally and decrease health care costs. We developed and evaluated a model of progressively implementing health-promoting schools with support from university medical school trainees in Canada and Uganda. The model uses oral health as a medium for establishing rapport and success around a topic with little stigma. The evaluation involved questionnaires of the Canadian trainees about practice intentions before and after involvement in the health-promoting schools to determine whether community-based learning in health-promoting schools resulted in more trainees planning to work in rural areas or underserved countries. We found that Canadian medical trainees cited their personal involvement and perceived ability to effect significant and identifiable positive change in both the school children and the community as reasons why they were more willing to practice in rural or under-served areas.

## 1. Introduction

One United Nations Millennium Declaration Goal states that “all people, everywhere, shall have access to a skilled, motivated, and well-facilitated health worker within a robust health system.”

However, this is still difficult to achieve for many rural and remote communities in well-developed nations like the United States of America [1], Australia [2], and Canada, and in developing nations, it is well-recognized that emigration of medical school graduates severely compromises human resources for health care [3]. In these developing nations, adequate human resources for equitable health service delivery in rural areas remains only a dream. 

To achieve the Millennium Declaration goal, it will be necessary to ensure that medical school graduates include those who are interested in, trained for, and willing to pursue careers in under-served rural and remote communities in their own countries [3], and that some graduates from developed nations contribute to care in less developed countries.

There is clear evidence that, at least in developed countries, growing up in a rural area is the strongest predictor for medical students subsequently practicing in a rural area [2, 4]. Other factors that appear to affect this choice include expressed intent on entering the program to study family medicine, and exposure to rural community practice during training. The factors that affect the decision of medical graduates in developing countries to remain in their countries and practice in rural areas, and influence graduates from developed countries to practice in under-served areas or abroad, are not as well studied nor as well understood. It is becoming critical that medical schools in developing countries acknowledge and address the need to retain their graduates, and for medical schools in developed countries to supply graduates who will take on the challenge of practicing in rural and remote areas, and contribute to care in under-served countries.

We report our experience in Canada and Uganda of a community-based education model that incorporates trainee involvement in “health-promoting schools” (HPS). HPS were conceived by the World Health Organization [5], as a means of providing the information and support systems necessary for the world-wide changes in behavior that are needed to improve health globally and decrease health care costs. Such schools incorporate health topics and the teaching of healthy practices into their regular curriculum, and they benefit from direction and support by health care providers and educators [6, 7]. We believed that having direct contact with individual students, and helping empower them to make positive changes in their lives through participation in the health-promoting school, would enable medical trainees to witness the potential they have as individuals to have significant impact on a problem that otherwise seems too daunting.

In 2000, the University British Columbia Department of Pediatrics and the elders in a remote First Nations community in western Canada collaborated to develop a school-based oral health promotion program called “Brighter Smiles”. In this program the Health-promotion activities were deliberately developed progressively, through dialogue that recognized the principles of different ways of knowing [8], symbiosis (input from, and benefits to, both the community and the university team members), collaboration, respect, shared leadership, and community-set priorities and pace of change. A three-year evaluation indicated that “Brighter Smiles” was successful in improving oral health status, knowledge, and practices [9], and that, through the rapport established, the community actively involved participating trainees to address other health issues for their children, with the result that the community school became a “health-promoting school” according to the World Health Organization criteria. Health-promoting schools strive to create an environment of physical and mental health, both within the school and in the community, and by enabling medical trainees to experience living in and contributing to, this environment, the trainees have valuable learning opportunities and experiences [10]. Students and faculty at Uganda's Makerere University (MU) College of Health Sciences heard about the Brighter Smiles program and, in 2006, asked for the program to be adapted to a Ugandan context. Oral health had been identified by WHO as a significant world-wide health issue [11], and by others as an issue in Uganda [12, 13]. Four health-promoting schools based on WHO principles [14] were established in rural communities in 2006, and an urban community was added 2 years later [15].

From 2006 to date, the BSA program has involved all the MU dental students. The MU program graduates an average of 12 students annually. Trainees are involved in BSA early in their education, and many elect to return to the communities regularly as they become more senior. In addition to contributing to the delivery of health promotion topics within the schools, there is an ongoing research and evaluation component to which they contribute. The annual evaluation and research visits to the HPS communities are conducted jointly with Canadian team members as part of an international partnership between the Faculties of Medicine at MU and UBC. 

Another spin-off of the Brighter Smiles program at UBC is the Global Health Initiative (GHI). GHI is a student-driven initiative to promote global health knowledge and skills at UBC. GHI provides a series of workshops to prepare interested students for electives in remote areas of Canada and abroad (which include mentorship from students with project experience), and opportunities to participate in well-organized, sustainable, and ethical international partnerships (such as the Brighter Smiles Africa project). This initiative has increased awareness of global health amongst students and faculty and has provided the impetus for the revision of the UBC medical school curriculum to incorporate a global health focus. 

We report here on the effect of the program on the postgraduation practice locations of UBC medical trainees who participated in the program, and on the medical school curriculum at MU and UBC. We also provide preintervention assessment of the emigration intensions of students at MU College of Health Sciences, which includes physicians, nurses, dentists, pharmacists, and radiology technicians.

## 2. Materials and Methods

This study had the approval of the Behavioural and Clinical Research Ethics Review Boards at the University of British Columbia, and the Ethics Board at Makerere University. All questionnaires and evaluations were completed anonymously. 

 We hypothesized that

- the personal involvement and connection with individual children in rural schools associated with participation in delivery of the Brighter Smiles/health-promoting school model would increase medical trainees' interest in practicing in rural areas and/or their interest in working in a developing country;
- the program could be the basis for significant changes in the curricula of medical schools in both developed and developing country.

Data on the UBC medical residents attending the Canadian Brighter Smiles school were obtained through the office of the Pediatric Residency, and data on their evaluation of the program was obtained through residents' anonymous standardized evaluations which are completed by 100% of students at the end of every rotation. 

Data on UBC students who participated in the Brighter Smiles Africa program were obtained through documentation incorporated in anonymously completed annual evaluations of the program, and self-report. The evaluations included Likert-scaled questions rating educational value of the BSA program and learning opportunities in Uganda, as well as open-ended questions on what was most valued about participation in the BSA program, and what could be improved.

Data on MU students were obtained through anonymous annual written evaluations.

Information on the effect on the curricula at UBC and MU were documented over the duration of the project.

## 3. Results

**Figure 1:**
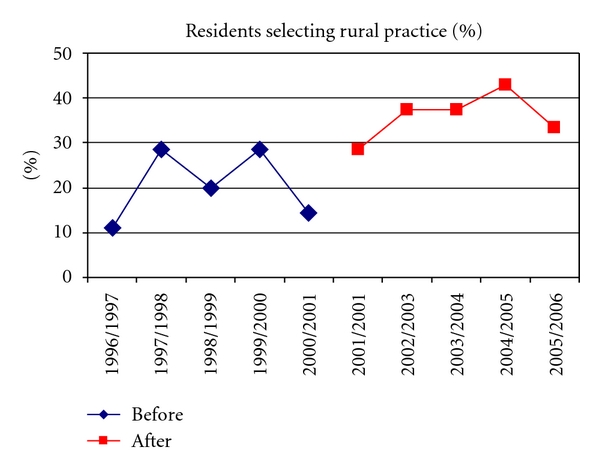
Percentage of pediatric residents choosing to practice in a small town or rural region following graduation before and after introduction of Brighter Smiles health-promoting school(s) in the residency program.

From 2001 to 2005, all residents (*n* = 36) in the Pediatric Residency program at UBC rotated through the remote aboriginal community at least once in order to contribute to the Brighter Smiles school program, 6 attended twice, and 4 attended 3 or more times over 5 years. Data on number and gender of trainees, and the numbers who chose small town or rural locations for their subsequent practices following graduation for the prior 5 years and these years are presented in Figure 1 and Table 1. Prior to the Brighter Smiles program, at least 50% of residents went on to specialty training, and, of the remainder, more than half chose to remain in large urban centers. 

For 4 of the 5 years, the “Brighter Smiles” rotation was the top-rated residency rotation; trainees placed particular value on the uniqueness of the experience and the range of learning opportunities. The Royal College of Physicians and Surgeons of Canada, in their 5-yearly accreditation assessment, identified the “Brighter Smiles” program as an innovative model and one of the major strengths of the UBC training program. 

The Brighter Smiles Africa (BSA) program involves approximately 6 UBC students each year. There have been 29 students in all, 5 of whom were neither medical nor dental students (economics, education, engineering, nutrition, and political science). All 29 attended the “Brighter Smiles” school in the First Nations community in preparation for their travel to Uganda. Of the 29 students, 12 (41%) have returned to Uganda, and 23 (79%) have mentored other students in the GHI program at UBC. Of the 24 medical-dental students, 10 (42%) have returned to Uganda. Seven of these students have since graduated from their programs, and 5 of these (70%) have chosen small town or rural practice.

Prior to the introduction of the BSA program at Makerere University, of the 92 MU students who responded to a question asking “Where do you hope to practice?” and given options of “Urban” or “Rural,” only 11 (12%) indicated that they planned a rural practice. Of the 12 dentistry students responding, none indicated that they planned a rural practice. On repeat survey after 3 years of the BSA program, 9 of 43 dental students (21%; *P* < .0001) indicated that they were planning to practice in a rural area.

At MU, the success of the BSA program has led the MU College of Health Sciences to introduce community-based education rotations where students now leave the urban medical school environment to live and work in a rural or underserved community for up to one month; 18 communities currently participate in this program. New students rotate with experienced fourth- or fifth-year students, and trainees return to the same community each year, so rapport and continuity evolve.

## 4. Discussion

It is critical to the health of the world's population that there is an equitable distribution of health resources, including the provision of health care workers to practice in what are often under-served rural areas. This applies to both developed and developing countries. More research is needed to establish what factors are conducive to practice in underserved areas [1, 2, 16, 17]. 

Although North American studies indicate that the primary predictor of practicing in a rural community is having grown up in a rural community, there are two significant factors that suggest that the desire to practice in a rural community should be instilled during training:

- the majority of trainees did not grow up in rural communities [1];
- this paradigm may not be applicable in all developing countries. In a previous anonymous questionnaire-based study, we obtained data (prior to involvement in community-based HPS learning) on MU medical trainees' intent to emigrate. We found that, for medical trainees at Makerere University, having grown up in a rural community is not predictive of future plans to practice in rural Uganda: the positive predictive value of growing up in a rural area, for plan to practice in a rural area, was 0.15, and the negative predictive value was 0.11.

Anecdotally, the impact on MU trainees of involvement in HPS programs via BSA has already been significant. At a workshop of BSA project stakeholders to review 5 years of program implementation, the deans of the Faculty of Medicine and College of Health Sciences, and the MU graduates, students, and faculty involved in BSA spoke about the educational value for the health professionals involved; the strength of the model as a means of health promotion; the value of the research and evaluation components; the merits of establishing more HP school sites in Uganda to increase child health promotion, and enable more trainees to be exposed to this form of community-based learning. 

If more training programs were to establish health-promoting schools following the Brighter Smiles model, in addition to the potential for positive impact on children's health, medical trainees in both developed and developing countries would experience community-based learning opportunities potentially conducive to careers in rural health. For some, this could provide the personal contact and involvement necessary to enable them to “see the one”, as advocated by mother Teresa:

“I never look at the masses as my responsibility; I look at the individual. I can only love one person at a time—just one, one, one. So you begin. I began—I picked up one person. Maybe if I did not pick up that one person, I would not have picked up forty-two thousand⋯. The same thing goes for you, the same thing in your family, the same thing in your church, your community. Just begin—one, one, one.”

If medical students are to fulfill their obligations as global citizens, they need curricula that provide the structured learning opportunities necessary to acquire the knowledge, skills, and experiences required to contribute effectively. Curriculum is the roadmap for providing tomorrow's professionals with these opportunities, and community-based learning is of particular relevance. Health-promoting schools, as WHO has identified, provide a model that can readily be adopted by others to foster the evolution of significant positive change amongst the children and the communities involved. Our evaluation of the involvement of health sciences trainees in the activities of such schools in Canada and Uganda suggests that the experience has a positive impact, which also has the potential to affect participants' career choices regarding rural or global health practice. Written evaluations indicate that all MU students value the unique knowledge and experiences gained through participation in the program in rural communities. Some are now considering rural practice activities as a career option and intend to help establish other health-promoting schools. Many wish they had been able to spend more time in the schools and provide more education to parents and community leaders. Collaborative learning with international partners is also valued, as are the joint opportunities to annually evaluate the impact of BSA, conduct research, and present and publish results. 

We have also shown that Health-promoting schools are not just for underdeveloped nations but have significant potential as training grounds in rural North American communities. 

Our experience represents medical trainees from different disciplines, including nursing, pharmacy, dentistry, general medicine, and pediatrics. The positive impact that is described across this wide range of disciplines suggests that the model would also be appropriate for trainees in family practice.

**Limitations**

The principle limitation of this study is the fact that there are other factors (such as government policy, world economy, and societal expectations) that affect both the types of students who choose and are admitted to medical and dental training programs, and the students' postgraduation intentions. These factors may have had influence in the same direction as our intervention (the BSA health-promoting schools program). Nevertheless, we believe that the BSA program is the primary factor driving the changes we report.

## 5. Conclusions

The Brighter Smiles model for establishing health-promoting schools was proven successful in rural communities in Canada and Uganda. In addition to addressing child-health issues relevant to the UN development goals, there are apparent benefits from the involvement of health care trainees in the activities of such schools. The community-based experiences gained through participation enable trainees to understand locally identified health priorities and see the impact of their involvement on the children and on the community as a whole. Canadian medical trainees who have participated in delivery of the Brighter Smiles program are more likely than others to practice in small towns or rural communities, and to undertake work in a developing country. Amongst Ugandans involved in the program, ongoing evaluation of their emigration and rural practice choices is warranted, as preliminary data suggests their attitudes are changing from those of the group originally surveyed, where the majority anticipated emigrating and few were considering rural practice.

## Figures and Tables

**Table 1:** Pediatric resident postgraduation rural practice choice showing five years prior, to and five years after, the introduction of Brighter Smiles health-promoting school in the residency program.

	Year	Female	Male	Small town/rural practice
Prior to Brighter Smiles	1996/1997	6	3	1
	1997/1998	3	4	2
	1998/1999	7	3	2
	1999/2000	3	4	2
	2000/2001	4	3	1

With Brighter Smiles	2001/2001	4	3	2
	2002/2003	8	—	3
	2003/2004	5	3	3
	2004/2005	3	4	3
	2005/2006	4	2	2
